# Barriers to Gestational Diabetes Management and Preferred Interventions for Women With Gestational Diabetes in Singapore: Mixed Methods Study

**DOI:** 10.2196/14486

**Published:** 2020-06-30

**Authors:** Sumali Hewage, Jananie Audimulam, Emily Sullivan, Claudia Chi, Tong Wei Yew, Joanne Yoong

**Affiliations:** 1 Saw Swee Hock School of Public Health National University of Singapore Singapore Singapore; 2 Integrated Health Promotion Unit Ministry of Health Transformation Office Singapore Singapore; 3 Family Planning 2020 United Nations Foundation Washington, WA United States; 4 Department of Obstetrics and Gynecology National University Hospital Singapore Singapore; 5 Division of Endocrinology Department of Medicine National University Hospital Singapore Singapore; 6 Center for Economic and Social Research University of Southern California Los Angeles, CA United States

**Keywords:** gestational diabetes, pregnancy, telemedicine, self-management, patient-centered care, mobile phone

## Abstract

**Background:**

Gestational diabetes mellitus (GDM) is associated with risks for both the mother and child. The escalated prevalence of GDM because of obesity and changes in screening criteria demands for greater health care needs than before.

**Objective:**

This study aimed to understand the perception of patients and health care providers of the barriers to GDM management and preferred interventions to manage GDM in an Asian setting.

**Methods:**

This mixed methods study used a convergent parallel design. Survey data were collected from 216 women with GDM, and semistructured interviews were conducted with 15 women and with 8 health care providers treating patients with GDM. Participants were recruited from 2 specialized GDM clinics at the National University Hospital, Singapore.

**Results:**

The patients were predominantly Chinese (102/214, 47.6%), employed (201/272, 73.9%), with higher education (150/216, 69.4%) and prenatal attendance at a private clinic (138/214, 64.2%), already on diet control (210/214, 98.1%), and receiving support and information from the GDM clinic (194/215, 90.2%) and web-based sources (131/215, 60.9%). In particular, working women reported barriers to GDM management, including the lack of reminders for blood glucose monitoring, diet control, and insufficient time for exercise. Most women preferred getting such support directly from health care providers, whether at the GDM clinic (174/215, 80.9%) or elsewhere (116/215, 53.9%). Smartphone apps were the preferred means of additional intervention. Desirable intervention features identified by patients included more information on GDM, diet and exercise options, reminders for blood glucose testing, a platform to record blood glucose readings and illustrate or understand trends, and a means to communicate with care providers.

**Conclusions:**

A GDM-focused smartphone app that is able to integrate testing, education, and communication may be a feasible and acceptable intervention to provide support to women with GDM, particularly for working women.

## Introduction

### Background

Gestational diabetes mellitus (GDM) is a well-established risk factor for type 2 diabetes mellitus (T2DM). In parallel with the growing T2DM epidemic [1], an increasing number of pregnancies are being complicated by GDM [2]. The International Diabetes Federation reported that GDM currently affects 1 in 6 births globally [3]. GDM is defined as the onset or first diagnosis of high blood glucose concentrations during pregnancy, which usually resolves after childbirth [4]. GDM demands drastic lifestyle changes in pregnant women and additional medical attention to minimize detrimental fetal and maternal outcomes [5,6].

As with other types of diabetes, women with GDM receive medical advice on appropriate nutrition alongside directions to perform self-monitoring of blood glucose and to administer insulin therapy if required [5-7]. For optimal blood glucose control, women must be engaged in an intensive process from diagnosis until the baby’s delivery, which usually spans 10 to 12 weeks. In conventional care, patients are expected to attend clinic appointments frequently so that health care providers can monitor glucose concentrations and patient behaviors closely. Patients often experience difficulties in adopting the required behavioral changes in a brief period, which can significantly compromise blood glucose control [8]. This is challenging for both patients and health systems and leads to exhausted capacities and burnout [9,10]. These challenges include patient barriers such as lack of reliable information, family and employment responsibilities, and social support and health system barriers such as lack of access to health care and inconsistent care delivery [11].

Asian populations are at a disproportionately higher risk of T2DM [12-15]. The prevalence of GDM in Singapore is above the global prevalence of 13.8% [3]. According to an analysis conducted with pregnant women who participated in a birth cohort study, *Growing Up in Singapore Towards Healthy Outcomes*, compared with high-risk GDM screening, universal screening diagnosed a significantly greater number of GDM cases (18.9% versus 9.8%, respectively) [16]. In addition, an analysis of the cost-effectiveness of GDM screening in the same study population showed that compared with no screening and high-risk screening, universal screening is cost-effective to reduce maternal and fetal complications due to GDM [17]. These results suggest that when it comes to policy implementation considerations for GDM care, universal screening has been shown to be significantly effective compared with high-risk screening, which is the current practice for GDM screening in Singapore [18].

It is widely accepted that patients’ active collaboration with health care providers and their appropriate behavior changes are key factors in optimal GDM management [19,20]. As a result, acknowledging the patient’s contribution is important in building a healthy patient-provider relationship. In addition, the US Institute of Medicine committee on quality of health care in America has identified patient-centered care as 1 of the 6 attributes of health care quality [21]. Therefore, focusing on patient-centric care has been the focus of providing quality health care.

### Objectives

In response to the anticipated increased prevalence from universal screening, to make improvements in the health care system to serve the needs of women diagnosed with GDM, it is important to understand the patients’ requirements. Therefore, this feasibility study explored 2 aims to understand (1) women’s perceptions of knowledge and management of GDM and (2) women and care providers’ perceptions and attitudes toward GDM control, related barriers, and potential interventions to overcome the barriers.

## Methods

### Design and Study Population

This study was undertaken with pregnant women who were diagnosed with GDM at the National University Hospital (NUH) in Singapore. Currently, all public hospitals in Singapore that provide prenatal medical care follow the latest World Health Organization (WHO) guidelines [22] for the diagnostic criteria of GDM and provide universal screening for GDM to pregnant women. Screening is typically offered between 24 weeks’ and 28 weeks’ gestation. Women diagnosed with GDM attend a comprehensive multidisciplinary educational session to help equip them with the knowledge required to manage GDM during pregnancy.

**Figure 1:**
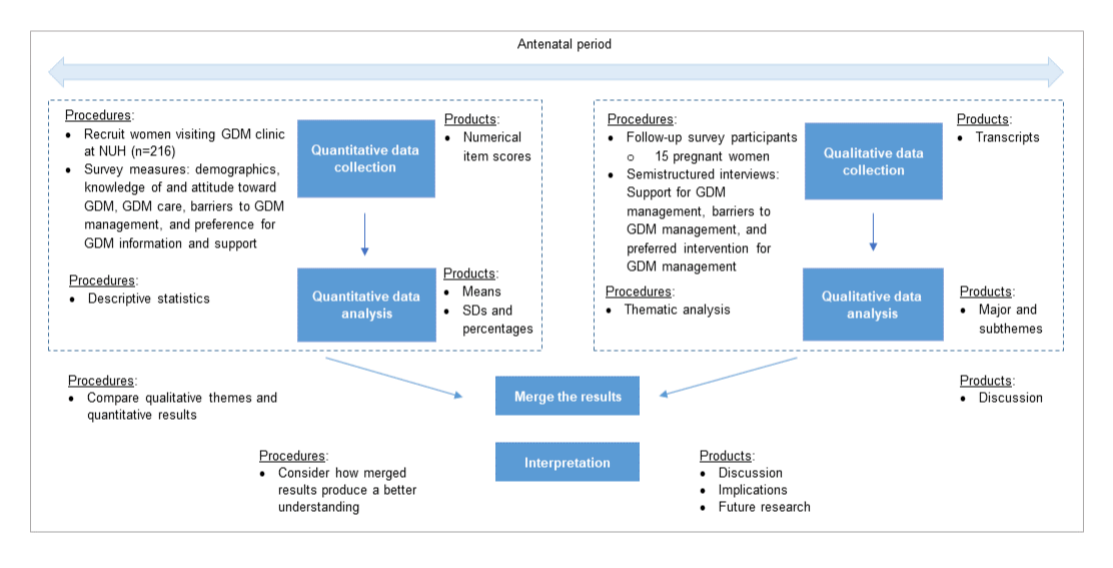
Convergent parallel mixed methods design. GDM: gestational diabetes mellitus; NUH: National University Hospital.

This study was conducted using mixed methods with quantitative and qualitative components and a convergent parallel design (Figure 1). A cross-sectional survey was undertaken in pregnant women attending 1 of 2 specialized GDM care clinics at NUH.

Inclusion criteria were being pregnant, aged 21 to 40 years, diagnosed with GDM during the index pregnancy, and attendance at the GDM clinic’s workshop on GDM management (delivered by a nurse educator and a dietitian). Exclusion criteria were inability to speak English and known type 1 or type 2 diabetes before the current pregnancy. Recruitment took place over 2 years between May 2015 and May 2017, with 2 phases of data collection.

### Study Tools

For the quantitative component, a 27-item cross-sectional survey (Multimedia Appendix 1) was used to collect demographic information, participant knowledge, control and attitude toward GDM, perceived barriers to GDM management, and current and preferred GDM support. Women were asked to rate their GDM knowledge before and after attending the GDM clinic, and the answer options included *excellent*, *good*, *fair*, *poor*. Questions related to GDM risk, perception, and attitudes were adapted from validated tools [23]. Attitude-related questions measured perceived personal control (2 questions), worry (2 questions), and optimism regarding recurrent GDM in future pregnancies (1 question). The 4 responses ranged from *strongly agree* to *strongly disagree*, and the scores were provided using a 4-point Likert scale (0-4). Average scores were calculated for each participant.

For the qualitative component, semistructured interviews were conducted using a topic guide based on published literature. This included questions on knowledge and attitudes regarding GDM, existing sources of support and coping mechanisms, current lifestyle practices, perceived barriers, ways of managing and monitoring GDM, and preferred intervention for GDM management.

Potential participants for the survey were approached in the waiting room of the GDM clinic. The study team refrained from approaching women who seemed tired or anxious. After getting informed consent, women were given a self-administered survey. No personal information was collected. In the first phase of the study, only a hard copy (paper) of the survey was offered. A web-based survey was added to the second phase to increase the recruitment rate.

The web-based form was created using the SurveyMonkey survey tool (SurveyMonkey Inc) and tested for any technical difficulties. The data collection was carried out using a closed survey. A total of 30 questions were distributed over 3 screens. Those who elected to complete the survey on the web were sent the link to the survey via email, and 3 additional *reminder* emails were sent to women in the following 6 weeks, inviting them to complete the survey before they were designated as true *nonresponders*.

For pregnant women who were willing to participate in prenatal interviews, sessions were scheduled based on their convenience. In the second phase, to increase survey completion, the women were given an incentive of $25 (US $17.5). In the first phase, 50 patients participated in the survey.

Of these 50 patients, 35 gave verbal consent to participate in the interviews and 15 participated in the interviews, and 2 study members (JA and SH) conducted all the interviews in English. Most interviews were conducted at the health care facility or at the participants’ homes, in a private space conducive for the participant to share thoughts effectively. Before the interview, participants provided informed consent, including consent for audio recording. Health care providers treating GDM patients from nursing, dietetics, and obstetrics and gynecology specialties were approached for interviews. The final number of interviews conducted was decided based on reaching thematic saturation as understood by the analysis, which was conducted in parallel with the data collection. Data saturation was reached at the 15th patient interview and the eighth care provider interview. Patient interview participants were from the first phase and were given Singapore $50 (US $35). Ethics approval was obtained from the National Healthcare Group (NHG) domain-specific review board (DSRB), Singapore (NHG DSRB Ref: 2015/00196).

**Figure 2:**
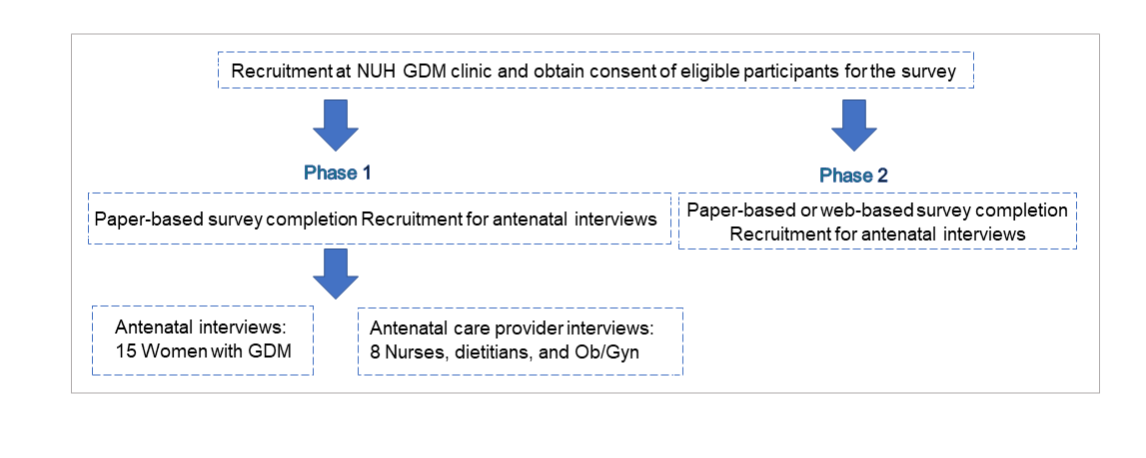
Study flow chart. GDM: gestational diabetes mellitus; NUH: National University Hospital; Ob/gyn: obstetrician/gynecologist.

The study flowchart outlines the steps involved in the recruitment, consent, and follow-up (Figure 2).

### Data Analysis

Univariate analysis was conducted to describe the study participants and GDM management. Results are presented using medians and IQRs for continuous variables with skewed distributions, whereas categorical variables are presented using frequencies and percentages. Perceived improvement in GDM knowledge after attending the GDM workshop was analyzed using the Fisher exact test. Those who received a score between 0 and 2 for GDM attitude-related factors, perceived GDM control, and perceived worry for GDM control were labeled as *low* and others were labeled as *high*. BMI was calculated using self-reported height and weight. The analysis was performed using the Stata statistical software (Stata Corp, 2013, Stata Statistical Software: Release 13).

The interviews were audio recorded, transcribed *verbatim*, and analyzed thematically [24]. The transcripts were not returned to participants for any further comments and/or corrections. First, transcription occurred verbatim, and the accuracy of the transcripts was verified by 1 of the researchers (SH). Following multiple readings of the verified transcripts, the main coder (SH) coded the transcripts using the Atlas Ti Software (QSR International Pty Ltd, 2012) and Microsoft Excel. Some themes were identified in advance, and some new themes were derived from the data. Reliability checks were performed by reading and checking transcripts, and a field expert (JY) verified the themes. The identified codes were used to derive the code structure that was needed to develop meaningful themes.

## Results

### Survey Completion

The average number of women attending the GDM clinic was 60 per month. In phase 1, the survey completion rate was 18.5%. In phase 2, 67.2% of the women visiting the clinic were interested in participating in the study, with 75.4% actually completing the survey, giving a completion rate of 44.0%. Most of the nonparticipants declined to participate without a reason, whereas some declined due to insufficient time. There were no significant differences between the 2 groups in terms of demographic characteristics (Multimedia Appendix 2).

### Demographic and Pregnancy Information

Table 1 provides the demographic and health information of the survey participants (n=216). In brief, the median age of the women was 32 years; 47.6% (102/216) women were Chinese, 73.9% (201/216) were in full- or part-time employment, and 69.4% (150/216) were university graduates. At the time of survey completion, the median duration of pregnancy was 30 weeks, and 50.4% (109/216) of women were multiparous, and 64.2% (138/216) of women attended a private clinic for their prenatal care. Approximately half (116/216, 53.9%) of the women were either overweight or obese before pregnancy and a large majority, 83.3% (180/216) women, perceived their health as *good* or *excellent*.

The women who participated in interviews had a demographic profile similar to that of the survey questionnaire participants. Half of the 15 participants in the age group of 31 to 35 years were primiparous or had a family history of T2DM, and most of them were Chinese or Indian, employed full time, with a degree, and attending a private prenatal clinic. Eight prenatal care providers at the GDM clinic were interviewed. Table 2 provides the demographic and health information of the patients interview participants and Table 3 shows the information of the health care provider interview participants.

**Table 1:** Survey participants’ demographic characteristics.

Variable		Values
Age (years) (n=209), median (IQR)		32 (22-40)
**Ethnicity (n=214), n (%)**		
	Chinese	102 (47.6)
	Malay	46 (21.5)
	Indian	43 (20.1)
	Other	23 (10.7)
**Employment (n=214), n (%)**		
	Full time	146 (68.2)
	Unemployed	55 (25.7)
	Part time	13 (6.1)
**Education (n=216), n (%)**		
	Degree or professional qualification	150 (69.4)
	Secondary education	62 (28.7)
	Lower than secondary education	4 (1.8)
Duration of pregnancy in weeks, median (IQR)		30 (8-39)
Parity—multiparous (n=215), n (%)		109 (50.4)
**Prenatal care (n=215), n (%)**		
	Private clinic	138 (64.2)
	Subsidized clinic	77 (35.8)
**Prepregnancy BMI (n=208), n (%)**		
	<23 kgm^−2^	96 (46.1)
	23-27.5 kgm^−2^	59 (28.4)
	>27.5 kgm^−2^	53 (25.5)
**Health (n=216), n (%)**		
	Poor	1 (0.5)
	Fair	35 (16.2)
	Good	158 (73.1)
	Excellent	22 (10.2)

**Table 2:** Interview participant profile—patients attending a gestational diabetes mellitus clinic (N=15).

Variable		Participants, n (%)
**Age (years)**		
	26-30	3 (20)
	31-35	8 (53)
	36-40	4 (27)
**Ethnicity**		
	Chinese	5 (33)
	Malay	3 (20)
	Indian	5 (33)
	Filipino and Sri Lankan	2 (13)
**Employment**		
	Full time	11 (73)
	Unemployed	3 (20)
	Part time	1 (7)
**Education**		
	Degree or professional qualification	14 (93)
	Secondary education	1 (7)
Parity—primiparous		8 (53)
**Prenatal care**		
	Private clinic	12 (80)
	Subsidized clinic	3 (20)
Family history of gestational diabetes mellitus		8 (53)

**Table 3:** Interview participant profile—health care providers serving at a gestational diabetes mellitus clinic (N=8).

Variable		Participants, n (%)
**Professional qualification**		
	Obstetrics and gynecology	2 (25)
	Diabetes care nurse educator	4 (50)
	Dietitian	2 (25)
**Years of total experience**		
	1 to 5	2 (25)
	6 to 10	3 (37)
	More than 10	3 (37)
**Gender**		
	Female	6 (75)

### Knowledge on Gestational Diabetes Mellitus

As reported by the participants, there was a significant improvement in perceived GDM knowledge after attendance at the GDM workshop (*χ*^2^_9_=54.0; *P*<.05). The vast majority, 93.5% of women, identified large for gestational age of the baby as a potential outcome of GDM, and 92% correctly identified the optimal range for pre- and postprandial capillary blood glucose levels.

As confirmed by the interviews, most of the women with a previous history of GDM were unaware or not fully aware of GDM before their clinical diagnosis. Those who had any knowledge of GDM mentioned that they had heard about it from peers with GDM. Most participants, even those with a family history of T2DM, felt that they did not have sufficient information on GDM. However, the participants felt that the clinic helped to *increase the understanding of GDM*, which is consistent with the survey results:

Actually, I didn’t know anything about it... So, I... thought that if you were diabetic then you kind of get it. But then I didn’t know something you can just develop during pregnancy as well. So, it was quite new to me.

ID_02

Now, the understanding level has gone high. I know like how to control and then how to manage my diet and then when to measure, what are the steps I need to take.

ID_13

### Management of Gestational Diabetes Mellitus

Almost all of the participants, 98.1% (210/214), controlled their diet; half, 48.1% (103/214), were physically active; and 21 participants, one-tenth, used insulin to regulate blood glucose concentrations. Most participants, 85.6% (185/216), were able to follow typical recommendations from the GDM clinic to perform finger prick blood testing 7 times a day for 2 days each week (Table 4).

Similarly, women who were interviewed also managed their blood glucose levels using diet modifications and monitored blood glucose levels using finger prick blood testing. Employed women, mostly Chinese and Malay, are more likely to consume outside food rather than home-cooked food. Few of the interviewed women were physically active, and only 2 used insulin therapy:

Mainly diet. I really watch my diet. Yes, actually now I, there’s only about a few choices that I can have every day.

ID_08

In addition, most women mentioned that they performed the test more frequently than required and mainly used test results to *interpret the effectiveness of GDM control* primarily diet. A few women believed that physical activity helped with blood glucose control:

So, from there I monitor. Let’s say one weekend and one weekday I monitor. But, let’s say if I want to feel, say in the morning I want to watch I just go on. So, it doesn’t matter, two times a week. Let’s say, watch three times or more, depends I feel want to check my sugar.

ID_09

**Table 4:** Management of gestational diabetes mellitus.

Variable		Value, n (%)
**Gestational diabetes mellitus management (n=214)**		
	Diet management	210 (98.1)
	Physical activity	103 (48.1)
	Insulin use	21 (9.8)
**Finger prick blood test (n=216)**		
	Record 7 readings on 2 days or more	185 (85.6)
	Most of the time	20 (9.3)
	Sometimes or never	11 (5.1)

### Attitudes Toward Gestational Diabetes Mellitus Control

In the survey, 97.7% of participants received a high score for perceived GDM control, whereas 74.5% had a high score for the perceived worry of GDM control. Most women (72.3%) perceived that they would not be able to control getting GDM in future pregnancies.

Consistent with the survey findings, at the time of the interview (subsequent to the clinic), many reported feelings of control related to diet and that they were *on the right track* in monitoring using the finger prick test:

That, I was just grappling around, reading on the internet, and then I was like “Oh my god, what do I do?” So, I was panicking.

ID_01

Because I am actually monitoring the sugar level at the moment. And I think it’s actually ok. So, far I am actually on the right track.

ID_06

Most women reported feeling worried or anxious when GDM was diagnosed, although the GDM clinic’s workshop helped to lessen their anxiety.

Health care providers also felt that most women were motivated to control their blood glucose levels and also stressed the importance of *discipline*, especially with respect to their food intake during this period:

Generally, this group of patients, they are very motivated. So, if they don’t have any language barriers, they should be able to understand that important for them... But in general, at least a good 80% of them seems to be quite receptive, I would say yeah...

ID_04_ Dietitian

### Barriers to Gestational Diabetes Mellitus Control

Most survey participants did not feel that they faced any significant barriers to GDM control. However, a significant minority (30.4%) felt they had difficulties in remembering blood testing schedules, whereas 22% felt discouraged because of the lack of immediate effects from their lifestyle changes (ie, continuing to have abnormal test results even after implementing GDM management). One-fifth of participants (21.5%) agreed or strongly agreed to experiencing difficulties following the recommended diet and physical activity plan. On the other hand, most women reported that they had sufficient help from family and friends and that family, cultural beliefs, or traditions did not interfere with GDM management (Table 5).

Conversely, based on the qualitative data, most employed women mentioned that they had barriers to GDM management mainly related to diet, increasing physical activity, and monitoring blood glucose levels. Women felt that they had a *limited (food) variety* because they had few food options that helped control blood glucose levels. These differences between quantitative and qualitative results may have been partly due to the lack of quantitative measures for assessing barriers to GDM control:

I have still maybe about three, four months or even more to go. So, I think I must expand the variety to make life easier.

ID_08

Only a few mentioned that they did exercise to control GDM. Although they had received advice to increase their physical activity, women felt unable to follow the recommendations mainly due to the *lack of time for exercise*:

I think one problem is that it’s not always very easy to exercise. Because one of the advice(s) is that you should have like a 10-15 minutes’ walk or some form of exercise after every meal. But it’s not very possible to do it after your lunch, for example, if you are working.

ID_02

According to the clinical practice guidelines of the Ministry of Health, Singapore, women are required to self-monitor their blood glucose levels [25]. At the NUH GDM clinic, women were advised to submit 7 blood glucose readings for 1 weekday and 1 weekend day each week. However, women had difficulty performing the required 7 readings for each testing day, largely because they forgot to take the test:

So, it is very hard to get these seven readings [finger prick test reading]. So, one thing is that it is tiring, and the other thing is that you, kind of unconsciously forget[s] with work and [a] lot [of] things as well. So, maybe you had your lunch and you finish[ed] it at one [pm] and then you take one [finger prick test] at three [pm]. And then you forget because you are like completely into your work.

ID_02

Similarly, health care providers reported that working women experienced more barriers to managing GDM than nonworking women:

Of course, if let’s say if they do work, then shift workers are quite difficult to tackle their meal timings and all. So, that is one of their barriers like limitation, their work commitments and all.[ID_04_ Dietitian]

**Table 5:** Barriers to management of gestational diabetes mellitus.

Barrier	Strongly disagree, n (%)	Disagree, n (%)	Agree, n (%)	Strongly agree, n (%)
Difficult to remember to take medication or blood tests at the scheduled times	28 (13.4)	117 (56.0)	59 (28.2)	5 (2.4)
Feel discouraged due to lack of immediate results (eg, high blood sugar)	23 (10.7)	144 (67.3)	45 (21.0)	2 (1.0)
Difficult to follow a specific diet and physical activity plan	28 (13.1)	140 (65.4)	41 (19.2)	5 (2.3)
Busy with family or work to manage my GDM properly	34 (16.0)	138 (64.8)	38 (17.8)	3 (1.4)
Nonspecific educational resources/opportunities about GDM	31 (14.6)	147 (69.0)	29 (13.6)	6 (2.8)
Family’s cultural beliefs/ traditional practices conflict with GDM management	69 (32.4)	125 (58.7)	17 (8.0)	2 (1.0)
Family and friends are not supportive of my efforts to eat right	74 (34.6)	127 (59.3)	12 (5.6)	1 (0.5)

### Support for Gestational Diabetes Mellitus Management

Of the 216 survey participants, 194 (90.2%) reported receiving GDM-related information from a physician, nurse, or trained counselor at the GDM clinic and 131 (60.9%) from websites. Moreover, 6.1% (16/216) of participants reported in-person support groups for expectant mothers as the method used least frequently (Table 6).

The interview themes appeared concordant with the survey results. Women were most likely to rely on health care providers to guide them with medical advice. Furthermore, almost all mentioned that they used the internet to obtain additional information related to GDM management. However, most of the resources used were intended for Western populations. Most of the women received help from their families and peers. In addition, they either approached peers with previous experience in controlling GDM or online forums. Most of them felt they had *sufficient support* in GDM management, whereas a few needed extra help:

Internet and friends and doctors.

ID_07

I would say mainly like my husband and I would say my colleagues.

ID_02

Well, one is you know because my colleague is also a pregnant woman and we are good friends, so we discuss a lot about it.

ID_14

The following interview results further support the above findings. According to health care providers, support from physicians, nurse educators, and dietitians was available in the usual clinic setting, and women were followed up fortnightly. On occasions requiring additional medical assistance, women were advised to approach the GDM clinic at the hospital. Some providers mentioned that they offered additional reading resources. In contrast, others pointed out the need to regulate the quality of supplementary material, especially of internet sources. Health care providers also acknowledged that, during this time, women may need additional emotional assistance from family members, including husbands and friends:

I mean we give them resources to read. The Royal College of Obstetricians and Gynecologists in the UK have lots [of] patient education leaflets that we can refer them to read at home and at leisure.

ID_02_Ob/gyn

And some of the database [are] based on different other countries like for an example [the] US. So, if you look at the US database, you realize that most of the calorie content, carbohydrate content slightly, maybe the portion size larger than us. So, that's why I always tell them, if you use a US database always to cut [portion size]. They need to reduce the amount of carbohydrate and amount [number] of calories.

ID_03_ Dietitian

**Table 6:** Source of gestational diabetes mellitus information.

Source	Value, n (%)
Doctor, nurse or trained counselor at the GDM^a^ clinic	194 (90.2)
Websites	131 (60.9)
Family members	66 (30.7)
Friends or colleagues	59 (27.4)
Web-based forums or support groups for new or expectant mothers	44 (20.5)
Doctor or nurse other than the GDM clinic	34 (15.8)
In-person support groups for expectant mothers	13 (6.0)

^a^GDM: gestational diabetes mellitus.

### Preferred Intervention for Gestational Diabetes Mellitus Management

In the survey, among the preferred methods of information and support for GDM management, the preferred option for most participants 80.9% (174/216) was *from a doctor, nurse or trained counselor at the GDM clinic*. Among their ranking, support from a physician or nurse outside of the GDM clinic ranked second (116/216, 53.9%), and this replaced the current support option of *websites*, which ranked as the third preferred option (46/216, 21.3%). However, only a minority of participants (34/216, 15.8%) were receiving this kind of care at the time of the survey (Table 6). The other listed options of family members, friends/colleagues, in-person support groups for expectant mothers, and support from other women who have previously managed GDM related to social support were less preferred.

Additional information regarding preferred interventions for GDM management was provided during the interviews. Most of the interview participants thought a smartphone app would be convenient*,* primarily because smartphones are now commonplace. They pointed out that such an intervention would be more helpful for GDM management, mainly for recording blood glucose readings daily, than a paper and pen, or a computer. In addition, women discussed the importance of having the means to *understand trends* and the steps that should be taken, if any, to rectify abnormal readings immediately:

I mean the most useful, convenient is the app [smartphone application]. Because everyone has a smartphone, and everyone can access.

ID_10

Maybe there’s an indication, your reading is good, there’s a comment to supplement your, because sometimes when you write down your reading, you do not know if it is on target, not on target, high risk, or low risk.

ID_11

Furthermore, the women stated the need of reminders for finger prick test schedules, general information about GDM, calorie calculations, and information about physical activity. Concerns about a possible app were relatively infrequently mentioned, but they included the level of complexity, technical glitches, and the need to charge the phone to access the app. Health care providers stressed the importance of strict control of blood glucose concentrations, and they also agreed that an app would be a convenient platform to assist GDM control. As additional features, health care providers suggested that the app could automate the transfer of blood glucose test results from the device to the provider overseeing the patient’s care. They felt that this step would increase the reliability of test results, including an automated reported option and the convenience of timely monitoring. According to them, the intervention would be effective only if it was user-friendly and affordable, especially for those of lower socioeconomic status:

They may forget to bring [blood glucose readings], that is what I was saying, they may forget to bring it [or] may not be accurate or they may not want to bring out to write it down. So, it will not be 100% accurate. (ID_05_nurse practitioner)

## Discussion

### Principal Findings

Our study was able to understand women’s and care providers’ perceptions on improving GDM care. Most women preferred the assistance of health care providers to control their blood glucose levels. Furthermore, the women, most of whom were employed, experienced barriers mainly due to limited reminders for monitoring blood glucose, difficulties in diet control, and inadequate time to be physically active. Smartphone apps appear to be preferred by women to assist the standard of care to better support blood glucose control. In addition, they anticipated that a *mobile app* can assist them to overcome their common difficulties as well to acquire reliable information on GDM and understand trends in blood glucose control.

One systematic review indicated that women with GDM feel overwhelmed in the initial period post diagnosis and that they are more likely to overcome these difficulties with appropriate medical assistance [26]. Our findings are consistent with these reported observations. GDM interventions have been shown to be important in helping women to curb adverse clinical outcomes as well as to elevate their quality of life [27]. These interventions appear to be successful due to receptiveness among highly motivated women who essentially want to safeguard their pregnancy.

According to our qualitative findings, the working women in the study reported experiencing barriers, including a lack of reminders for blood glucose monitoring, issues related to diet control, and lack of time for recommended exercise. Although physical activity has been shown to be effective in regulating blood glucose concentrations among women with GDM [28], pregnant women in Singapore are less likely to be active, especially in the later stages of pregnancy [28]. This lack of a behavioral change may be further augmented by other commitments, such as work-related responsibilities. Identified gaps in GDM management highlight the need for appropriate interventions to integrate into busy lifestyles.

The participants in this study reported receiving advice primarily from specialized health care providers or web-based resources currently. However, among their preferences, the women conveyed the need for further assistance from health care providers other than the specialized GDM clinic. This highlights that women prefer to rely on medical personnel for advice, although web and smartphone usage among pregnant women is a common phenomenon with a significant ability to influence their health behaviors [29]. In the present environment, readily available health-related information from a wide variety of nonmedical resources may increase the possibility of erroneous information. Therefore, it is important to get the assistance of health care providers to critically review web-based content for medical accuracy and suitability [29,30].

As demands for health care are increasing, many parts of health systems, including diabetes care, are seeking help from telemedicine [31]. As defined by the WHO, telemedicine involves providing health care, including information on diagnosis, treatment, and disease prevention, where distance is a critical factor [32]. Similarly, several intervention studies have been published on telemedicine solutions for women with GDM [33-36]. Two meta-analyses concluded that telemedicine interventions, primarily mobile apps, may conveniently replace face-to-face clinic visits between women and health care providers without compromising the quality of care [37-41]. However, both reports pointed out the limited number of such interventions and the need for further investigation of possible cost evaluations.

**Figure 3:**
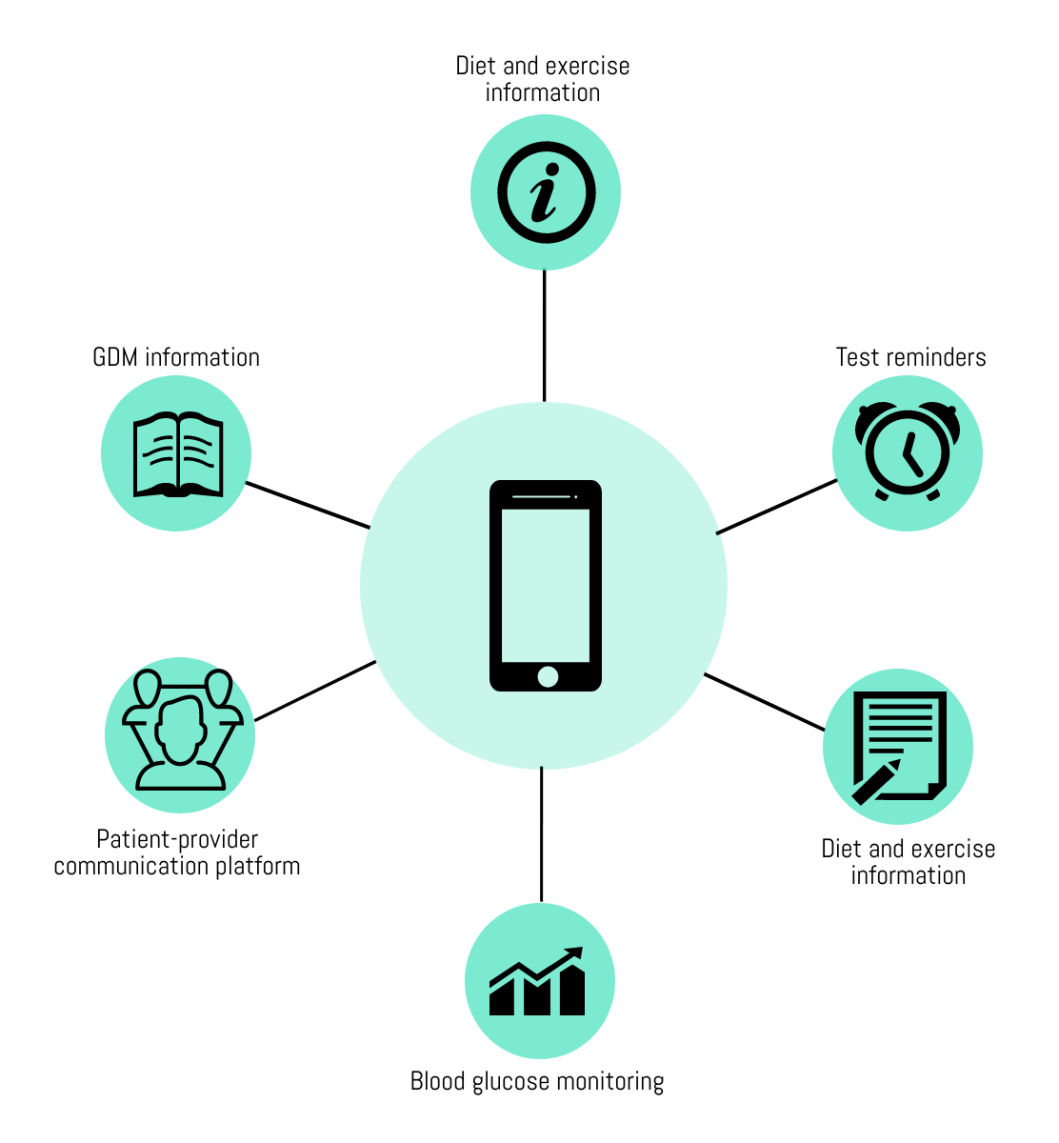
Preferred features of a smart phone app for gestational diabetes mellitus management. GDM: gestational diabetes mellitus.

In general, smartphone apps are considered to be patient-centric interventions [42]. As pointed out by study participants, a smartphone app would be a viable solution for most of the identified issues and could help improve lifestyle behaviors. The interventions undertaken included recording information on food, physical activities, blood glucose concentrations, and insulin regimens. To date, few studies have been conducted to understand the usability and acceptance of smartphone apps among women with GDM [37,38]. Neither of these reported studies assessed the perception and contribution of potential users before developing their programs. Having user input—both women with GDM and health care providers who treat women with GDM—is vital for the successful implementation of such interventions as it increases user acceptability and intervention sustainability (Figure 3) [43,44].

### Strengths and Limitations

The mixed methods approach used here has deepened our understanding of the needs that women with GDM have regarding GDM management. This study has a few limitations. Data collection was conducted in 1 public hospital, which may not be entirely representative of other centers in Singapore. However, in 2016, 85% to 90% of women in Singapore who were 25 to 34 years old were employed, and 56% of the women either had a degree, diploma, or professional qualification [45]. Our study sample has a similar demographic distribution; hence, the findings appear largely generalizable to women with GDM in Singapore. Second, we were not able to measure the change in participant knowledge before and after the GDM workshop or to evaluate the effectiveness of the information delivery via the workshop [46]. A further limitation was that only 1 researcher coded and analyzed the qualitative data. Although measures were taken to increase the response rate, the overall survey completion rate was low. In addition, only women who could speak and write English and were willing to participate were recruited. Therefore, it is possible that the results are biased toward those women who chose to participate. As suggested by a previously published meta-analysis, it is worth leveraging technology to facilitate behavior management and to make the intervention available to intended groups [37]. Recently, the National Health Service in the United Kingdom approved the use of a smartphone app in the management of GDM. It is anticipated that the intervention will result in fewer clinic visits for working women and reduce inconvenience and unnecessary workplace absenteeism [47]. In Singapore, most women of reproductive age are employed and may benefit from such technological advances. Due to the increasing demand in the health care sector, telemedicine options have been gaining attention as a feasible option. The contribution of users is critical when designing a technological intervention, especially for pregnant women [48]. Our study identified the barriers experienced by women with GDM. These gaps may be addressed with a smartphone app. This was the commonly agreed intervention by the women and care providers to assist in optimal GDM management while easing the pressure on the local health system.

### Conclusions

In conclusion, as informed by this study, a carefully planned randomized control trial is likely to be useful in assessing the effectiveness and cost-effectiveness of a smartphone app to minimize adverse maternal and fetal outcomes of GDM and the optimal use of health care resources in Singapore.

## Appendix

Multimedia Appendix 1 Cross-sectional survey questionnaire.

Multimedia Appendix 2 Participants’ characteristics by data collection phase.

## Abbreviations

DSRB: domain-specific review board
GDM: gestational diabetes mellitus
NHG: National Healthcare Group
NUH: National University Hospital
NUS: National University of Singapore
T2DM: type 2 diabetes mellitus
WHO: World Health Organization
