# Supplementary material for: Barriers to Gestational Diabetes Management and Preferred Interventions for Women With Gestational Diabetes in Singapore: Mixed Methods Study
Source: JMIR Form Res. 2020 Jun 30;4(6):e14486. doi: 10.2196/14486 (PMC7367517; doi:10.2196/14486)
Supplement: Multimedia Appendix 1 [file formative_v4i6e14486_app1.docx]

**Gestational Diabetes Research Survey**

**Introduction**

Dear Participant,

The aim of the research is to understand the barriers and facilitators in management for mothers recently diagnosed with **gestational diabetes mellitus (GDM)** in Singapore.

This questionnaire consists of related questions on:

1. Demographics and health related behaviors
2. Knowledge about Gestational Diabetes Mellitus
3. Constraints and problems in the management of **Gestational Diabetes Mellitus (GDM)**

There is no correct or best answer. Please answer the survey as honestly as you can.

Your decision to participate in this research is strictly voluntary. You can withdraw from the study at any time without giving any reasons and no responses will be collected. You do not have to answer any question that you do not wish to. Your identity remains anonymous.

Please contact Ms Jananie Audimulam at jananie@nuhs.edu.sg or Dr Joanne Yoong at ephysjy@nus.edu.sg if you have any questions about this survey.

*1. By checking this box, I hereby acknowledge that I have read and understood the terms of my participation in this survey, and I have agreed to take part in the above research.

Yes, I fully understand the terms and conditions

**Questionnaire**

1. How old are you? ______
2. What is your ethnicity?
3. Chinese
4. Malay
5. Indian
6. Others

1. What is the highest level of education you have completed?
2. No formal education c. GCE ‘O’/’N’ level e. Degree / Professional Qualification
3. PSLE d. GCE ‘A’ level / Dip
4. What is your current job status?
5. Full time work b. Part Time work c. Unemployed d. Student
6. What is your housing type?
7. HDB 1-3 rooms c. Executive flats and other public flats
8. HDB 4-5 rooms d. Condominium or Private Housing
9. Is this your first pregnancy?
10. Yes b. No
11. How far along you are in your current pregnancy? ________ weeks
12. Where do you go for your prenatal care?
13. Clinic G ( subsidized patient ) b. Women’s Clinic ( private patient) c. Jurong Clinic ( private patient)
14. What was your pre-pregnancy weight _____________kg and height ___________cm?
15. How would you rate your current state of health?
16. Excellent
17. Good
18. Fair
19. Poor
20. How would you rate your level of knowledge about gestational diabetes mellitus (GDM) before attending the gestational diabetes clinic?
21. Excellent
22. Good
23. Fair
24. Poor
25. How would you rate your level of knowledge about gestational diabetes mellitus (GDM) after attending the gestational diabetes clinic?
26. Excellent
27. Good
28. Fair
29. Poor
30. If a pregnant women has GDM, her baby may be: (circle all that apply)
31. Larger than usual c. Born early
32. Smaller than usual d. I do not know

1. Normal blood sugar levels are:
2. 4–8 mmol/l b. 7–15 mmol/l c. 2–10 mmol/l d. I do not know

1. The next few statements will provide important information about how people like yourself feel about GDM. Please check the answer that you think best reflects **your opinion and attitudes.**

|  | Strongly Agree | Agree | Disagree | Strongly disagree |
| --- | --- | --- | --- | --- |
| If I make a good effort to manage my GDM, I am much more likely to control it |  |  |  |  |
| I worry about managing my GDM |  |  |  |  |
| I am able to manage my GDM successfully on my own |  |  |  |  |
| If I am going to get GDM in future pregnancies there is not much I can do about it |  |  |  |  |
| Worrying about GDM is very upsetting |  |  |  |  |

1. What steps are you currently taking to manage your GDM? (circle all that apply)
   1. Diet control b. Insulin control c. Physical activity d. Other, please specify. _________________________________
2. Do you keep a record of your finger prick blood test results?
3. Yes, I record more than I am advised to do.
4. Yes, I record all seven readings on both days.
5. I record most of the time.
6. I record sometimes.
7. I never record.
8. Have you previously heard about taking an OGTT (Oral glucose tolerance test) after delivery?
   1. Yes, it has been recommended for me by my doctor / in the GDM clinic
   2. Yes, from my friends / family or other sources
   3. No, I have never heard about this
9. How important do you think it is for you to take an OGTT (Oral glucose tolerance test) after delivery?
10. Very important
11. Somewhat important
12. Not very important
13. Not important
14. I don’t know
15. Are you intending to make a follow up visit to repeat the OGTT after delivery as part of your post-partum care?
16. Yes

b. Maybe

c. No

1. There are many possible barriers / constraints that people face when considering GDM control during pregnancy, some of which are listed below. Please tell us how important the following potential barriers are to you by checking a square in each row that Please choose the option that best reflects **your opinion and attitudes.**

|  | Strongly Agree | Agree | Disagree | Strongly disagree |
| --- | --- | --- | --- | --- |
| I lack the knowledge and understanding to make and follow a specific diet and physical activity plan |  |  |  |  |
| I lack the knowledge and understanding of medications, side effects, schedules and adjustments to comply with my medication |  |  |  |  |
| I often feel discouraged due to lack of immediate results (e.g.; high blood sugar). |  |  |  |  |
| Educational resources/opportunities about GDM are inconvenient or non-specific |  |  |  |  |
| I am too busy with family or work to manage my GDM properly |  |  |  |  |
| My family and friends are not supportive of my efforts to eat right. |  |  |  |  |
| It is difficult to remember to take medications or blood tests at the scheduled times. |  |  |  |  |
| My family’s cultural beliefs or traditional practices conflict with aspects of the recommended GDM diet management plan |  |  |  |  |

Other please specify ______________________________________________________________________________________

1. Where do you **currently** get information and support related to GDM management or control? Please circle all that apply.
   1. Doctor , nurse or trained counselor at the GDM clinic
   2. Doctor or nurse other than the GDM clinic
   3. Family members
   4. Friends/colleagues
   5. Websites
   6. In-person support groups for expectant mothers
   7. Online forums / support groups for new or expectant mothers
   8. Others, specify _________________________________________________________
2. **Ideally,** **from whom** would you most prefer to get information and support related to GDM management or control? Please rank all the options below from 1 (most preferred) to 7 (least preferred)

|  |
| --- |
|  |
|  |
|  |
|  |
|  |
|  |
|  |

- 1. Doctor , nurse or trained counselor at the GDM clinic
  2. Doctor or nurse other than the GDM clinic
  3. Family members
  4. Friends/colleagues
  5. Websites
  6. In-person support groups for expectant mothers
  7. Online forums / support groups for new or expectant mothers
  8. Others, specify ______________________________________

Unlike GDM, Type 2 diabetes is a chronic condition in which there is a high level of sugar in the blood. Type 2 diabetes is the most common form of diabetes

1. How would you rate your current level of knowledge about Type 2 diabetes?
   1. Excellent
   2. Good
   3. Fair
   4. Poor

1. What do think your own risk or chance is for getting Type 2 diabetes over the next 10 years?
   1. A high chance
   2. A moderate chance
   3. A slight chance
   4. Almost no chance
2. The next few statements will provide important information about how people like yourself feel about Type 2 diabetes. Please check the answer that you think best reflects **your opinion and attitudes.**

|  | Strongly Agree | Agree | Disagree | Strongly disagree |
| --- | --- | --- | --- | --- |
| I feel that I have little control over risks to my health. |  |  |  |  |
| If I am going to get diabetes, there is not much I can do about it. |  |  |  |  |
| I think that my personal efforts will help control my risks of getting diabetes. |  |  |  |  |
| People who make a good effort to control the risks of getting diabetes are much less likely to get diabetes. |  |  |  |  |
| Compared to other women of my same age, I am less likely than they are to get diabetes. |  |  |  |  |
| Compared to other women of my same age, I am less likely than they are to get a serious disease. |  |  |  |  |

1. Please use this space if you have any comments.

________________________________________________________________________________________________________________________________________________________________________________________________________________________________________________________________________________________________________________________________________________________________________

If yes, please leave your contact information to the survey administrator. We will be in touch with you shortly.

Thank you for participating in this survey!
