# Supplementary material for: Barriers to Gestational Diabetes Management and Preferred Interventions for Women With Gestational Diabetes in Singapore: Mixed Methods Study
Source: JMIR Form Res. 2020 Jun 30;4(6):e14486. doi: 10.2196/14486 (PMC7367517; doi:10.2196/14486)
Supplement: Multimedia Appendix 2 [file formative_v4i6e14486_app2.docx]

| **Supplementary Table 1**: Participant’s characteristics by phase of the data collection | | | |
| --- | --- | --- | --- |
| Variable | Phase 1 | Phase 2 | p-value |
| Age, years mean (SD) (n=209) | 32.79 (4.15) | 31.76 (3.92) | 0.118 |
| Ethnicity (n=214) |  |  | 0.096 |
| Chinese | 19 (38) | 83 (50.6) |  |
| Malay | 9 (18) | 37 (22.6) |  |
| Indian | 16 (32) | 27 (16.5) |  |
| Other | 6 (12) | 17 (16.3) |  |
| Employment (n=214) |  |  | 0.122 |
| Full-time | 29 (59.2) | 117 (70.9) |  |
| Non-full-time | 20 (40.8) | 48 (29.1) |  |
| Education (n=216) |  |  | 0.251 |
| Degree | 38 (76) | 54 (32.5) |  |
| Less than degree | 12 (24) | 112 (67.5) |  |
| Prenatal care (n=215) |  |  |  |
| Private clinic | 34 (69.4) | 104 (62.6) | 0.387 |
| Subsidized clinic | 15 (30.6) | 62 (37.4) |  |
| Pregnancy in weeks, mean (average) | 29.96 (5.91) | 30.5 (5.6) | 0.556 |
